# A Bibliometric Analysis of Metabolic Reprogramming in the Tumor Microenvironment From 2003 to 2022

**DOI:** 10.1002/cnr2.2146

**Published:** 2024-08-19

**Authors:** Yupeng Xi, Rui Liu, Xing Zhang, Qiujun Guo, Xiwen Zhang, Zizhen Yang, Honggang Zheng, Qingqiao Song, Baojin Hua

**Affiliations:** ^1^ Department of General Internal Medicine, Guang'anmen Hospital China Academy of Chinese Medical Sciences Beijing China; ^2^ Department of Oncology, Guang'anmen Hospital China Academy of Chinese Medical Sciences Beijing China; ^3^ Department of General Internal Medicine Xi'an Fifth Hospital Xi'an Shanxi China

**Keywords:** bibliometric analysis, immunotherapy, metabolic reprogramming, tumor microenvironment, tumor‐infiltrating immune cells

## Abstract

**Background:**

Despite considerable progress in cancer immunotherapy, it is not available for many patients. Resistance to immune checkpoint blockers arises from the intricate interactions between cancer and its microenvironment. Metabolic reprogramming in tumor and immune cells in the tumor microenvironment (TME) influences anti‐tumor immune responses by remodeling the immune microenvironment. Metabolic reprogramming has emerged as an important hallmark of tumorigenesis. However, few studies have focused on the TME and metabolic reprogramming. Therefore, we aimed to explore the current research status and popular topics in TME‐related metabolic reprogramming over a 20 years using a bibliometric approach.

**Methods:**

Studies focusing on metabolic reprogramming and TME were searched using the Web of Science Core Collection database. Bibliometric and visual analyses of the articles and reviews were performed using Bibliometrix, VOSviewer, and CiteSpace.

**Results:**

In total, 4726 articles published between 2003 and 2022 were selected. The number of publications and citations has increased annually. Cooperation network analysis indicated that the United States holds the foremost position in metabolic reprogramming and TME research with the highest volume of publications and citations, thus exerting the greatest influence. Among these institutions, Fudan University displayed the highest level of productivity. *Frontiers in Immunology* showed the highest degree of productivity in this field. Ho Ping‐Chih made the most article contributions, and Pearce Edward J. was the most co‐cited author. Four clusters were obtained after a cluster analysis of the authors' keywords: TME, metabolic reprogramming, immunometabolism, and immunity. Immunometabolism, glycolysis, immune cells, and tumor‐associated macrophages are relatively recent keywords that have attracted increasing attention.

**Conclusions:**

A comprehensive landscape of advancements in metabolic reprogramming and the TME was evaluated, which provided crucial information for scholars to further advance this promising field. Further research should explore new topics related to immunometabolism in the TME using a transdisciplinary approach.

**Abbreviations**
ACAT1: acetyl‐CoA acetyltransferase 1
CTLA‐4: cytotoxic T‐lymphocyte‐associated protein 4
ICBs: immune checkpoint blockages
IDO1: indoleamine‐2,3‐dioxygenase 1
PD‐1: programmed cell death receptor
PD‐L1: programmed cell death‐ligand 1
TME: tumor microenvironment
TLS: total link strength
TAMs: tumor‐associated macrophages
Tregs: CD4+ regulatory T cells
TIL‐Bs: tumor‐infiltrating B lymphocytes
WoSCC: Web of Science Core Collection

## 1 Introduction

Cancer ranks at the top among noncommunicable diseases, presenting the highest mortality rates globally [1]. In 2020, the International Agency for Research on Cancer's Global Cancer Statistics 2020 report revealed that approximately 19.3 million new cases of malignant tumors and nearly 10 million deaths worldwide were attributed to cancer [2]. With ongoing advancements in research, traditional radiotherapy and chemotherapy have transitioned to a new era of targeted therapy and immunotherapy [3]. Among these advancements, immunotherapy was recognized by “*Science*” as one of the “Top 10 Breakthroughs of 2013.” Immunotherapy has shown significant and durable clinical efficacy in some refractory cancers and has been widely used to treat several cancer types. However, owing to the complexity of the tumor microenvironment (TME) and numerous factors that affect the response to immunotherapy, the clinical efficacy of immunotherapy in certain tumors needs improvement [4]. The TME is a dynamic and complex network in which various components including effector immune cells, inhibitory immune cells, and stromal components are involved in increased tumor cells proliferation and invasion, development of drug resistance, and reduction of anti‐tumor immunity at different tumor stages [5, 6]. Therefore, there is an urgent need to study the interaction mechanisms between TME and immunotherapy, improve the responsiveness of patients to immunotherapy, and restore or enhance the anti‐tumor immune response.

Owing to uncontrolled growth and proliferation, tumor cells frequently rely heavily on a reprogrammed metabolic state to adapt to stress and sustain innate proliferation [7]. Unlike normal cells, which primarily rely on oxidative phosphorylation for energy production, tumor cells tend to favor glycolysis even in the presence of oxygen, a phenomenon known as the “Warburg effect” [8]. In addition to increased glucose uptake and aerobic glycolysis, other metabolic pathways involving lipids and amino acids are often reprogrammed, resulting in dysregulated nutrient depletion and oncometabolite accumulation in the TME [9]. Emerging evidence shows that metabolic alterations in tumor cells have a commensurate influence on the abundance and effector function of immune cells, and jointly affect the effectiveness of the anti‐tumor immune response [10, 11]. Therefore, metabolic reprogramming is considered an important hallmark of cancer and can promote the survival, proliferation, and metastasis of tumor cells. Metabolic reprogramming processes are critical in tumorigenesis and progression [12, 13, 14]. Understanding the intricate metabolic reprogramming that governs tumor‐associated immune cells is of utmost importance for deciphering the underlying mechanisms and enhancing the efficiency of immunotherapy in combating cancer. Therefore, the metabolic reprogramming of tumor and immune cells in TME has become a hot topic in oncology research. Currently, numerous approaches outline the development of an academic field systematically, among which bibliometric analysis is one of the most prevalent [15]. Bibliometrics is a statistical analysis method used to quantitatively analyze research hotspots and characteristics of scientific output in a specific field [16]. This method has been widely used to analyze the productivity of researchers, journals, countries, and institutions in the medical field, and can offer crucial information and facilitate further exploration of new study directions [17, 18]. Using a bibliometric approach, Wu, Deng, and Zhao [19] assessed research hotspots and future directions of immunotherapy‐TME studies. Ai et al. [20] analyzed the current status, worldwide trends, and upcoming directions in the field of T‐cell lipid metabolism. To our knowledge, no bibliometric analysis focusing on the global status of metabolic reprogramming and the TME has been published. Therefore, the present study aimed to comprehensively analyze the research progress with respect to metabolic reprogramming in the TME using the Web of Science database. Through a comprehensive analysis of the research themes and emerging hotspots, we hope to provide guidance for future research and promote progress in the field.

## 2 Materials and Methods

### 2.1 Data Source and Search Strategy

**FIGURE 1:**
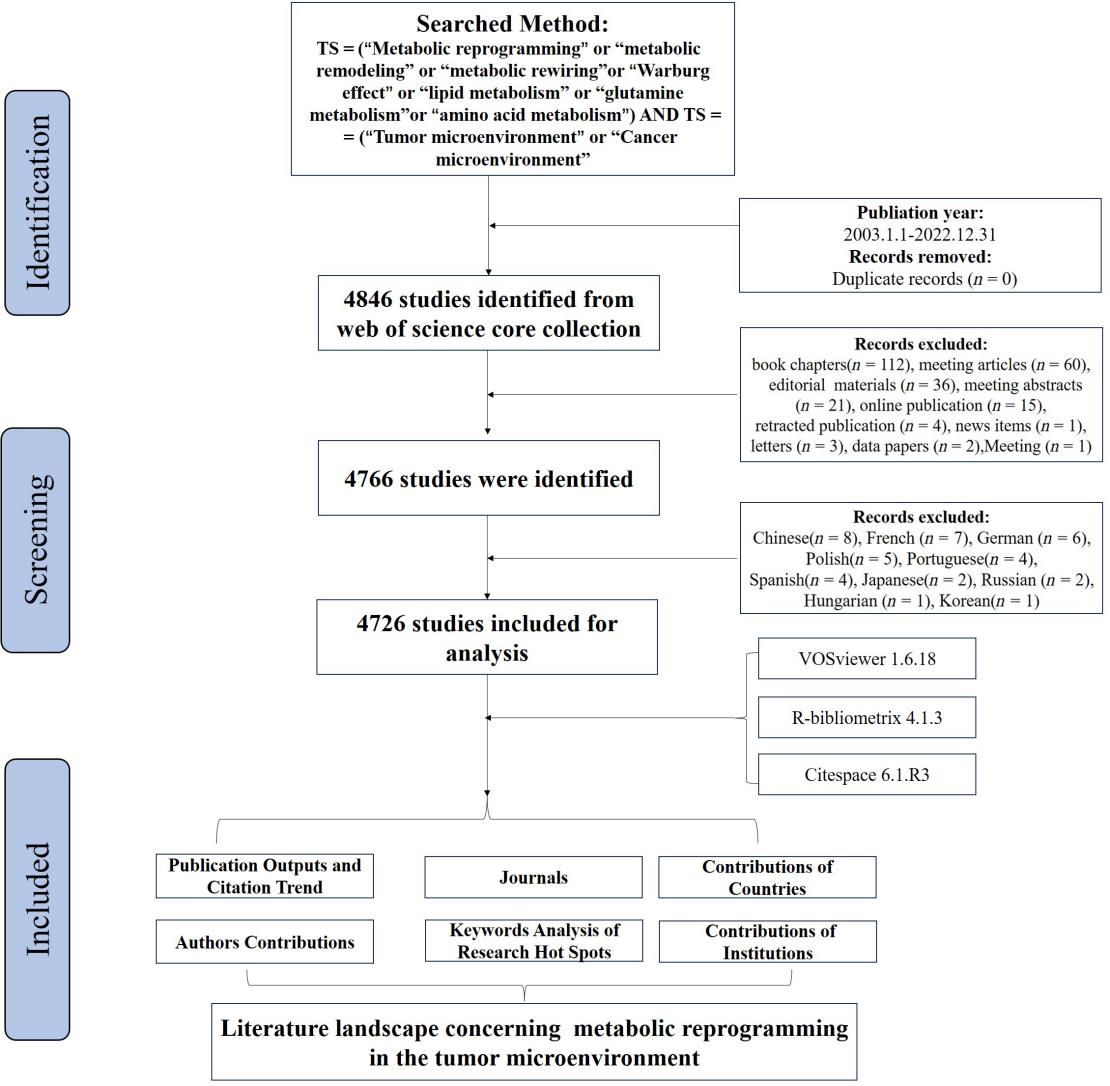
Flow chart of the literature selection process.

We utilized the Web of Science Core Collection (WoSCC) as the primary data source for information on metabolic reprogramming and TME over the past 20 years (2003–2022). To prevent citation fluctuations resulting from frequent database updates, we conducted literature retrieval on a single day (August 15, 2023). The search terms were as follows: topic: (TS = Metabolic reprogramming) OR (TS = Metabolic remodeling) OR (TS = Metabolic rewiring) OR (TS = Reprogramming of metabolism) OR (TS = Metabolic alterations) AND (TS = Tumor microenvironment) OR (TS = Microenvironment, Tumor) OR (TS = Microenvironments, Tumor) OR (TS = Tumor Microenvironments) OR (TS=Cancer Microenvironment) OR (TS=Cancer Microenvironments) OR (TS = Microenvironment, Cancer) OR (TS = Microenvironments, Cancer); period: (from 2003 to 2022); literature types: (articles or reviews). The language used was restricted to English. Although several articles on the Warburg effect were available before 2003, relatively few have specifically proposed a correlation between tumor metabolism and the TME. Consequently, we considered this timeframe to represent an evolving pattern in our area of interest. All documents related to metabolic reprogramming and TME were exported in “full records and references” format. The R packages “bibliometrix,” VOSviewer, and CiteSpace were then retrieved and imported for a comprehensive bibliometric analysis. A comprehensive workflow design for literature screening and data analysis is shown in Figure 1.

### 2.2 Data Analysis and Visualization

R (version 4.1.2), R‐bibliometrix 3.0.3 package (https://www.bibliometrix.org) CiteSpace (version 5.7. R5) and VOSviewer software (version 1.6.19) were used to analyze the collected data and generate visual graphs [21]. VOSviewer software [22] was used to visualize the bibliometric network, including the co‐citation network analysis of articles, co‐citation network of journals, collaboration among countries and institutions, author's collaboration network, co‐citation network of authors, and keyword co‐occurrence maps. Clusters are denoted using nodes of the same color, with larger node sizes indicating a higher number of publications, whereas the thickness of the links represents the strength of the interconnections between the nodes. The overall level of cooperation was evaluated quantitatively using a total link strength (TLS). The Bibliometrix package, a tool based on the R language, was used to visualize data analysis and social network graphs [23]. We used the robust R package ggplot2 (version 3.4.2) to visualize the results. Using this tool, we analyzed productive journals, countries, institutions, and word clouds based on authors' keywords as well as countries' contributions and collaborations. CiteSpace [24], a commonly used bibliometric analysis and visualization software, was used to construct a timeline graph and perform burst analysis of the co‐occurring keywords.

## 3 Results

### 3.1 Analysis of Publication Outputs and Citation Trends

**FIGURE 2:**
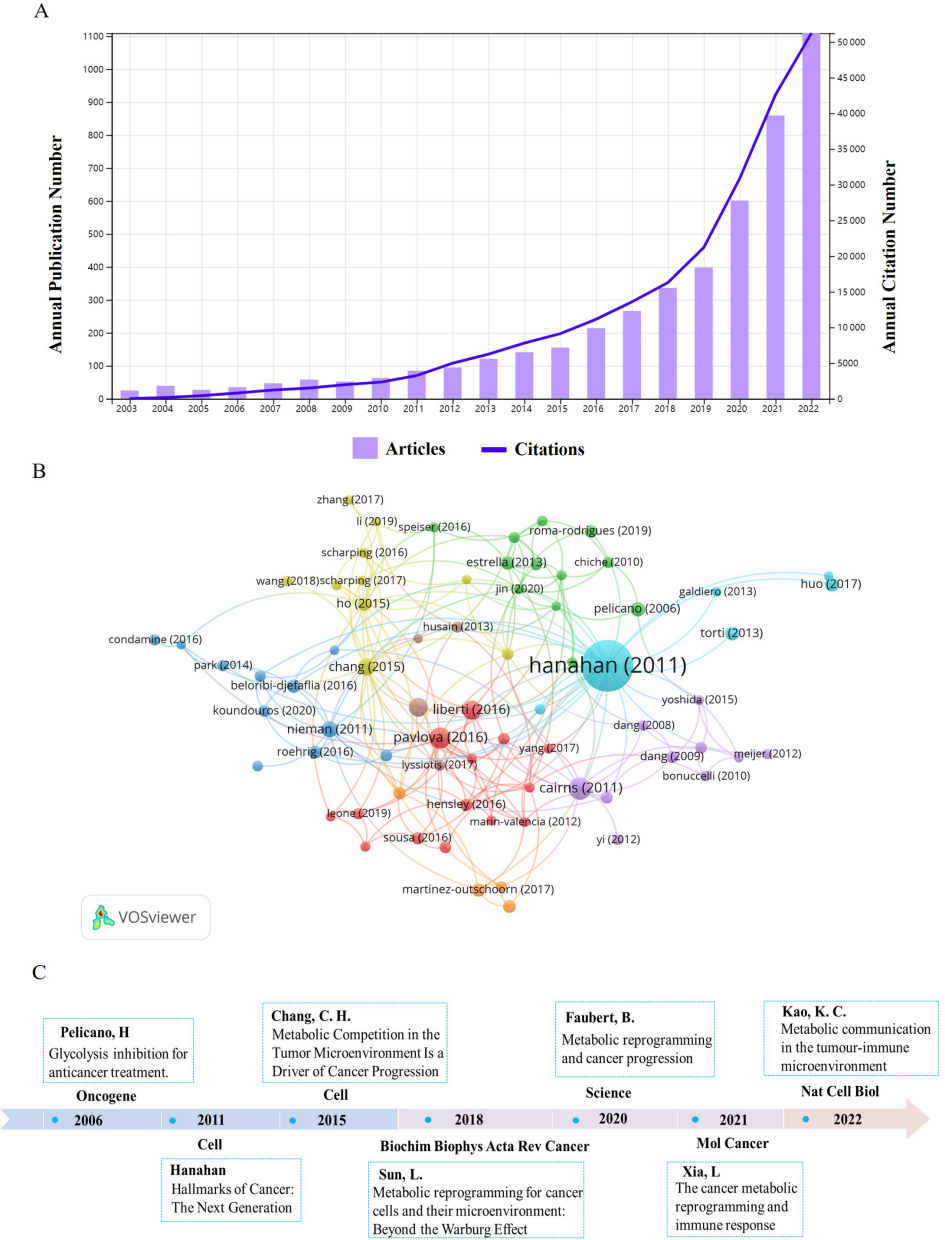
Overall growth trends in annual publications and analysis of top‐cited articles. (A) The number of publications and citation trends related to metabolic reprogramming and TME for each year from 2003 to 2022; (B) co‐citation network analysis of articles using VOSviewer; (C) timeline of top‐cited articles on metabolic reprogramming and TME research.

A total of 4726 publications, comprising 2827 original research articles and 1899 reviews associated with metabolic reprogramming in the TME, were screened from the WoSCC database (Figure 1). Overall, there is a gradual increase in the annual number of publications over time. A line graph illustrating the changes in the literature on metabolic reprogramming and the TME from 2003 to 2022 was generated (Figure 2A). The entire period can be categorized into three phases based on the annual publication growth rate: the first phase (2003–2011), second phase (2012–2016), and third phase (2017–2022). In 1924, German physiologist Otto Heinrich Warburg discovered that glucose metabolism was significantly elevated in tumor cells despite the presence of enough oxygen [8]. He received the Nobel Prize in Physiology or Medicine in 1931 for discovering the nature and mechanism of action of respiratory enzymes [25]. In 2011, Hanahan and Weinberg [26] proposed that metabolic reprogramming is a hallmark of carcinogenesis. Relatively few articles were published between 2003 and 2011; however, from 2012 to 2016, several articles on metabolic reprogramming were published, with a few studies focusing on the correlation between metabolic reprogramming and the TME. In 2015, several studies reported the phenomenon of metabolic reprogramming in the TME and its implications for tumor immunotherapy. Since then, the number of articles related to metabolic reprogramming and TME has suddenly increased. A total of 3569 articles were published between 2017 and 2022, accounting for 76% of all literature. These findings indicate an increasing interest in the role of metabolic reprogramming in the TME in recent years.

According to the co‐citation network analysis using VOS viewer (Figure 2B), the article “Hallmarks of Cancer: The Next Generation” was published in the journal *Cell* in 2011 (IF = 64.5) and has been cited 42 519 times [26]. The second most‐cited article was “Regulation of cancer cell metabolism” by Cairns, Rob A. in *Nature Reviews Cancer* (IF = 78.5) [27]. Table 1 presents the ranking of the 10 articles with the highest number of citations. By assessing the total citation count and its relevance to metabolic reprogramming in the TME, a series of important articles were systematically selected to completely comprehend the research progress on metabolic reprogramming in the TME. A chronological timeline was constructed to highlight the important milestones characterized by these significant articles (Figure 2C).

**TABLE 1:** Top 10 cited articles on metabolic reprogramming and TME from 2003 to 2022.

Rank	Title	First author	Journal	Year	Total citations
1	Hallmarks of Cancer: The Next Generation	Hanahan D	*Cell*	2011	42 519
2	Regulation of cancer cell metabolism	Cairns RA	*Nature Reviews Cancer*	2011	2646
3	The biology and function of fibroblasts in cancer	Kalluri R	*Nature Reviews Cancer*	2016	2268
4	Metabolic Competition in the Tumor Microenvironment Is a Driver of Cancer Progression	Chang CH	*Cell*	2015	1773
5	Metabolic reprogramming in macrophages and dendritic cells in innate immunity	Kelly B	*CELL RESEARCH*	2015	950
6	Lipid metabolic reprogramming in cancer cells	Beloribi‐Djefaflia S	*Oncogenesis*	2016	835
7	Lactate: A Metabolic Key Player in Cancer	Hirschhaeuser F	*Cancer Research*	2011	725
8	Mitochondrial metabolism and cancer	Porporato PE	*Cell Research*	2018	632
9	Targeting Tumor Microenvironment for Cancer Therapy	Roma‐Rodrigues C	*International Journal of Molecular Sciences*	2019	618
10	Revisiting the hallmarks of cancer	Fouad YA	*American Journal of Cancer Research*	2017	591

### 3.2 Analysis of Journals

**FIGURE 3:**
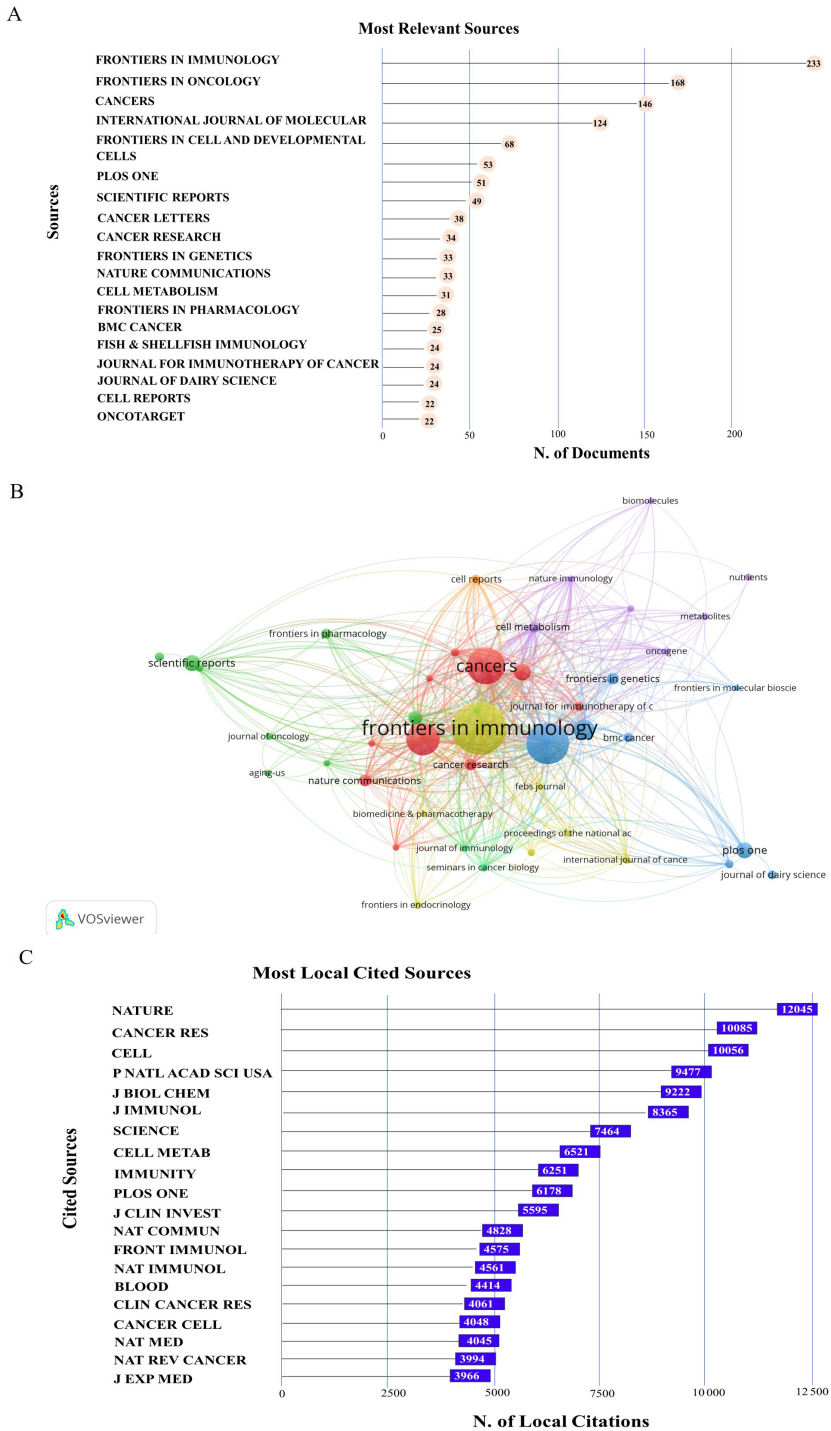
Analysis of journals. (A) Top 20 most productive journals on metabolic reprogramming and TME; (B) co‐citation network of journals by using VOSviewer; (C) top 20 most cited journals.

Articles on metabolic reprogramming in the TME between 2003 and 2022 were published in 586 academic journals. Table 2 shows a compilation of the top 15 academic journals that published a substantial number of articles related to metabolic reprogramming and TME. These journals collectively represented 23.57% (1114 of 4726) of the total publications in this field. The journal *Frontiers in Immunology* exhibited the highest degree of productivity with 233 publications (Table 2 and Figure 3A/B). This was followed by 168 articles in the journal *Frontiers in Oncology* and 146 publications in the *Cancers*. Of the most cited journals (Figure 3C), the top three were *Nature* (12 045 articles), *Cancer Research* (10 085 articles), and *Cell* (10 056 articles).

**TABLE 2:** Top 15 productive journals in metabolic reprogramming and TME from 2003 to 2022.

Rank	Journal	Total number of publications	Total citations	Impact factor 2022	JCR partition
1	*Frontiers in Immunology*	233	7589	7.3	Q1
2	*Frontiers in Oncology*	168	3146	4.7	Q2
3	*Cancers*	146	2292	5.2	Q2
4	*International Journal of Molecular Sciences*	124	3083	5.6	Q1
5	*Frontiers in Cell and Developmental Biology*	68	708	5.5	Q2
6	*Cells*	53	860	6.0	Q2
7	*Plos One*	51	1698	3.7	Q2
8	*Scientific Reports*	49	974	4.6	Q2
9	*Advance in Experimental Medicine and Biology*	41	967	3.65	Q2
10	*Cancer Letters*	39	1485	9.7	Q1
11	*Cancer Research*	34	2916	11.2	Q1
12	*Frontiers in Genetics*	33	88	3.7	Q2
13	*Nature Communications*	33	1965	16.6	Q1
14	*Cell Metabolism*	31	3848	29	Q1
15	*Frontiers in Pharmacology*	28	583	5.6	Q1

### 3.3 Analysis of Countries and Institutions

**FIGURE 4:**
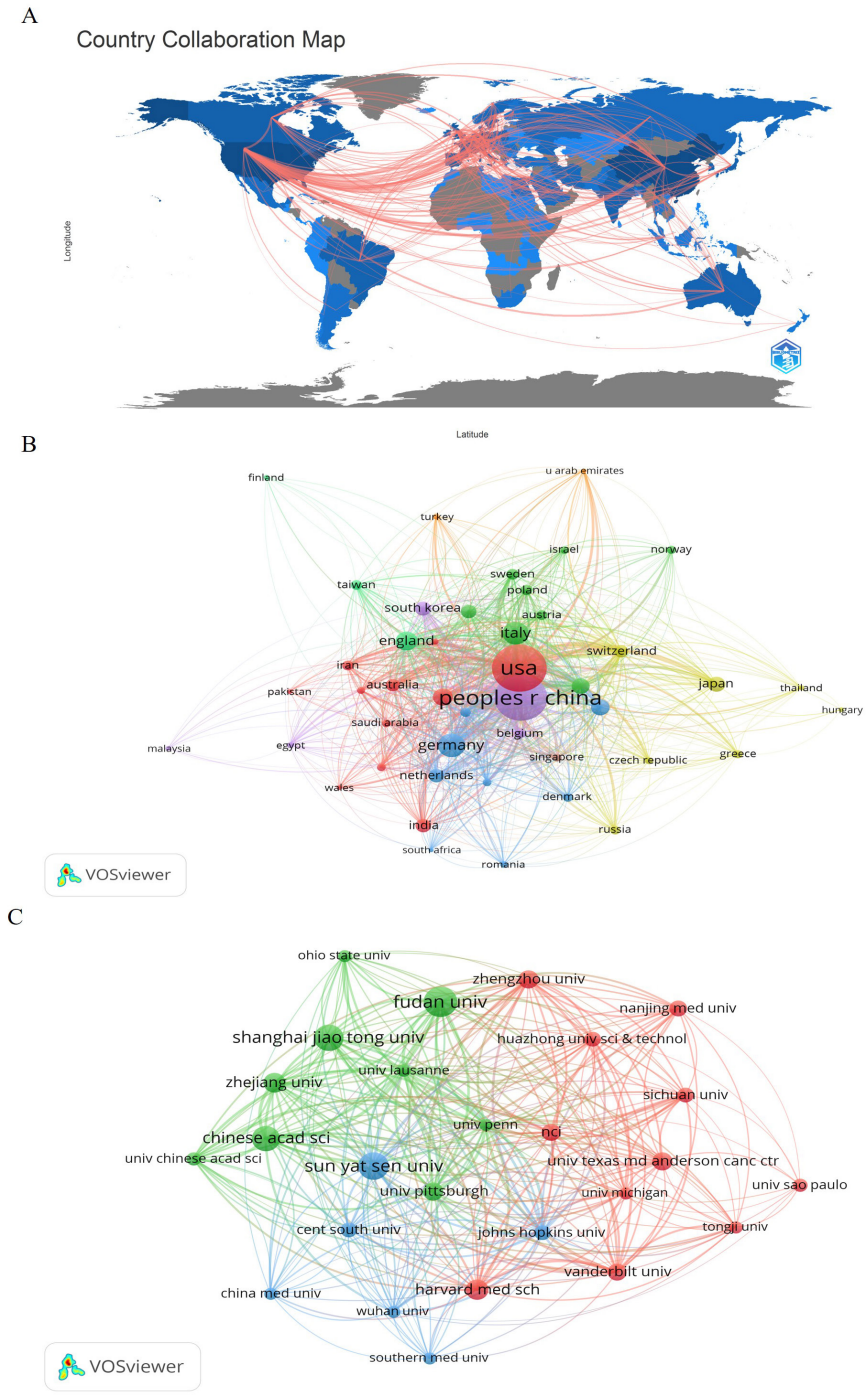
Analysis of countries and institutions. (A) Countries' contribution and collaboration in metabolic reprogramming and TME using R‐bibliometric; (B) countries' contribution and cooperation map using VOSviewer; (C) institutions contribution and cooperation map using VOSviewer.

The number of publications and total citations of the 10 most productive countries are presented in Table 3. The United States was the most productive country with 1491 articles, followed by China (1410), Germany (371), Italy (333), and England (236). The number of citations of published articles reflects a country's academic influence in the field. The United States and Switzerland were prominent contributors to this area of research, with citation counts of 150 472 and 49 324, respectively, surpassing other countries such as China (31 259), Germany (25 190), and Italy (16 796). The rapid development of metabolic reprogramming and TME in the United States has increased its influence through closer cooperation and innovative studies with other countries. Collaboration maps involving 94 countries on metabolic reprogramming and TME (Figure 4A,B). The results reflect the close collaboration and exchanges between the United States, China, and Western European countries. In addition, the United States had the strongest international collaboration network (TLS = 10 219) and cooperated closely with the others.

**TABLE 3:** Top 10 productive countries working on metabolic reprogramming and TME from 2003 to 2022.

Rank	Country	Total number of publications	Percentage	Total citations	H‐index
1	USA	1491	31.55	150 472	158
2	China	1410	29.83	31 259	79
3	Germany	371	7.85	25 190	70
4	Italy	333	7.05	16 796	63
5	England	236	4.99	14 256	56
6	France	199	4.21	10 951	50
7	Spain	172	3.64	7217	46
8	Canada	165	3.49	11 483	56
9	Japan	151	3.20	7084	45
10	South Korea	124	2.62	4026	31
11	Brazil	120	2.54	4716	36
12	India	120	2.54	2967	24
13	Netherlands	114	2.41	6371	43
14	Australia	104	2.20	7139	39
15	Switzerland	103	2.18	49 324	42

These published articles were contributed by 4646 institutions worldwide. The institutions with the highest productivity in terms of metabolic reprogramming and TME are presented in Table 4; 90% (18 institutions) of the top 20 institutions were located in China (10, 50%) and the United States (8, 40%). The remaining 10% (two institutions) were located in Brazil and South Korea. Fudan University published the most significant quantity of articles (208), followed by Sun Yat‐sen University (175), Shanghai Jiao Tong University (128), Central South University (127), University of Pittsburgh (125), Zhejiang University (118), and Vanderbilt University (116). The collaboration network shows that the most influential institutions were the University of Lausanne (288 TLS), University of Pennsylvania (246 TLS), and Chinese Academy of Sciences (241 TLS; Figure 4C).

**TABLE 4:** Top 10 most productive institutions regarding metabolic reprogramming and TME from 2003 to 2022.

Rank	Institution	Country	Total number of publications	Total citations	Total link strength	H‐index
1	Fudan University	China	208	1921	209	25
2	Sun Yat Sen Univ	China	175	2282	150	22
3	Shanghai Jiao Tong Univ	China	128	2090	174	23
4	Cent South Univ	China	127	1355	241	20
5	Univ Pittsburgh	USA	125	3332	210	25
6	Zhejiang Univ	China	118	1028	178	18
7	Vanderbilt Univ	USA	116	3456	216	31
8	Univ Texas MD Anderson Canc CTR	USA	111	10 486	101	43
9	Univ Michigan	USA	109	2635	123	21
10	Harvard Med Sch	USA	104	13 937	191	46

### 3.4 Analysis of Author Contributions

**FIGURE 5:**
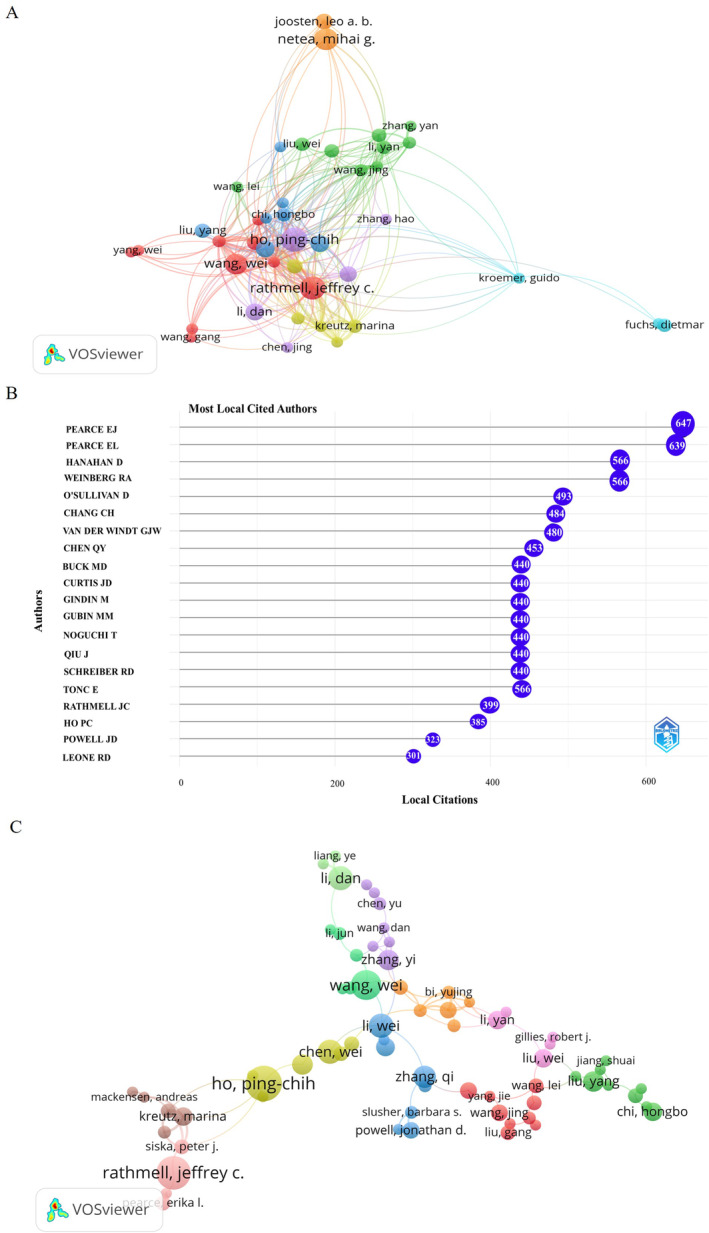
Analysis of authors. (A) Co‐citation network of authors with the most publications using VOSviewer; (B) top 20 cited authors for research focusing on metabolic reprogramming and TME; (C) visualization map of author's cooperation network using VOSviewer.

In total, 24 156 scholars were involved in publications related to the fields of metabolic reprogramming and the TME, with an average of 5.1 authors per article. The co‐citation network of the authors with the most publications (Figure 5A). Ho Ping‐Chih, from the University of Lausanne, published the largest number of articles [21]. In 2019, an article titled “Navigating metabolic pathways to enhance anti‐tumor immunity and immunotherapy” published in *Nature Reviews Clinical Oncology* showed that an acidic and nutrient‐deprived TME can exert metabolic stress on infiltrating immune cells, leading to a suppressive immune response and tumor immune evasion [28]. This study has been cited 425 times. Rathmell, Jeffrey C. (18 articles) and Netea, Mihai G. (17 articles) followed closely. Pearce Edward J. had the highest number of citations (Figure 5B). To identify key authors and investigate cooperation patterns, the collaboration network map was visualized using a VOS viewer (Figure 5C). Within the collaboration network, Ho Ping‐Chih had the strongest collaborative intensity with other scholars, followed by Jeffrey C. Rathmell.

### 3.5 Keyword Analysis of Research Hot Spots

**FIGURE 6:**
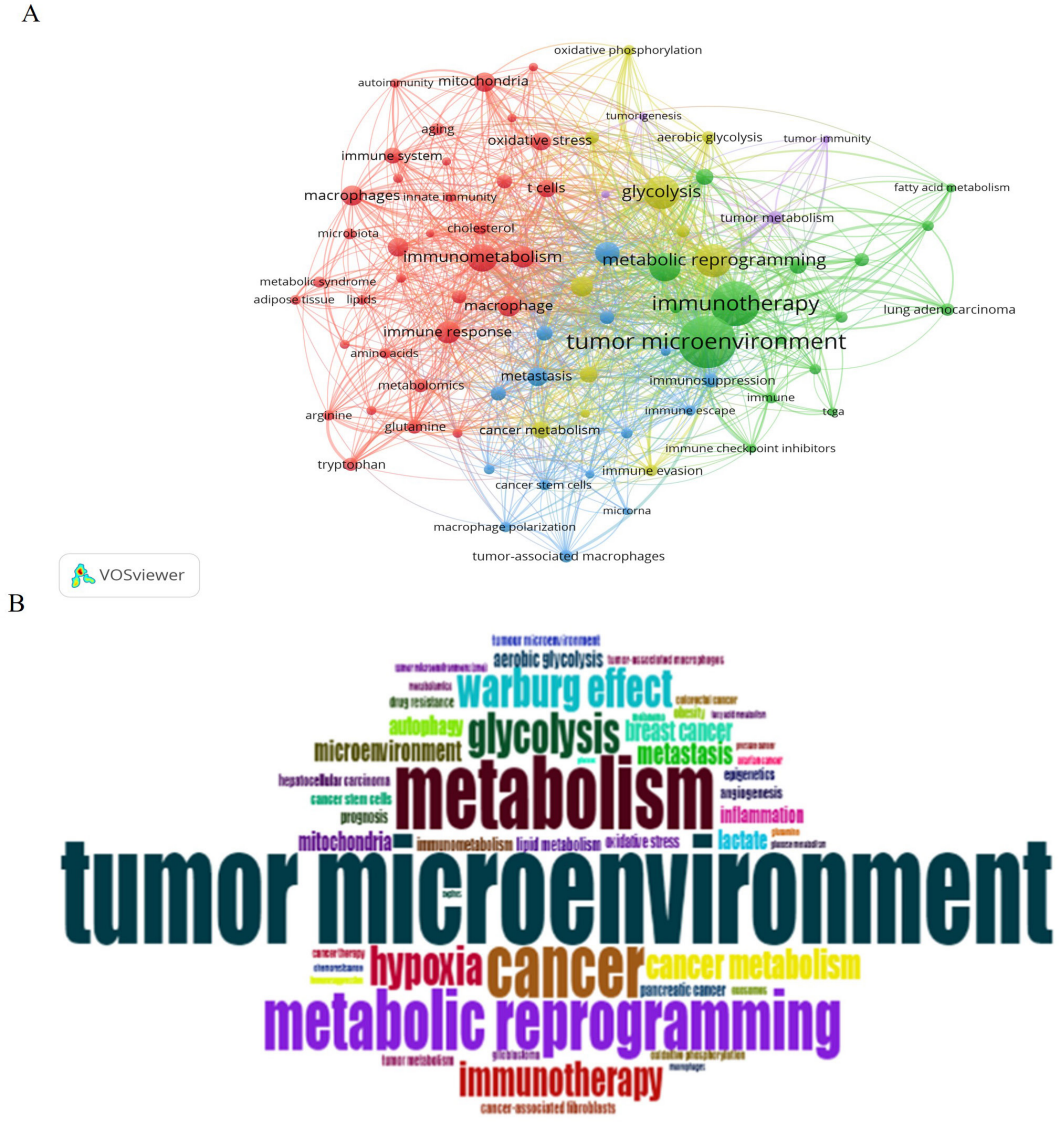
Analysis of author's keywords. (A) The co‐occurrence visualization network of the author's keywords using VOSviewer; (B) word cloud based on the author's keyword using biblioshiny.

A keyword co‐occurrence analysis is a frequently employed method for identifying prevalent topics. Figure 6A illustrates the network and overlay visualization maps of the co‐occurring keywords. The 10 most frequently used keywords were TME, immunotherapy, glycolysis, metabolic reprogramming, lipid metabolism, immunometabolism, immune response, hypoxia, immunity, and macrophages. All the keywords were grouped into four clusters (Figure 6A and Table 5). The first node with the highest TLS in the green cluster was related to the TME and immunotherapy, keywords such as “tumor microenvironment,” “tumor immune microenvironment,” “immunotherapy,” “lipid metabolism,” “glucose metabolism,” and “amino acid metabolism” are included in this analysis. The second cluster was yellow and related to metabolic reprogramming and glycolysis, including keywords such as “glycolysis,” “Warburg effect,” “oxidative phosphorylation,” “tumor microenvironment,” and “T cell.” The third group was red and related to immunometabolism and gut microbiota, including keywords such as “immunity,” “immunology,” “immunomodulation,” “lipids,” “microbiome,” “gut microbiota,” “lipopolysaccharide,” “macrophage,” and “cytokines.” The blue cluster was associated with immune and extracellular vesicles, involving keywords such as “immune cells,” “immunosuppression,” “tumor‐associated macrophages,” “immune escape,” “extracellular vesicles,” “exosomes,” “microrna,” and “cancer stem cells.” Similarly, the frequency of each keyword is visually represented using a word cloud (Figure 6B). The term “tumor microenvironment” appeared most frequently, being mentioned 425 times, followed by “metabolism” (289 times), “cancer” (243 times), and “metabolic reprogramming” (236 times).

**TABLE 5:** Main author's keywords cluster on metabolic reprogramming and TME from 2003 to 2022.

Rank	Green cluster	Yellow cluster	Blue cluster	Red cluster
1	Tumor microenvironment	Glycolysis	Hypoxia	Immunometabolism
2	Immunotherapy	Metabolic reprogramming	Immune cells	Immune response
3	Lipid metabolism	Warburg effect	Immunosuppression	Macrophages
4	Glucose metabolism	Cancer metabolism	Angiogenesis	Oxidative stress
5	Ferroptosis	Lactate	Tumor‐associated macrophages	T cells
6	Immune infiltration	T cell	Macrophage polarization	Gut microbiota
7	Amino acid metabolism	Cancer immunotherapy	Immune escape	Microbiota
8	Immune microenvironment	Immune evasion	Exosomes	Lipids
9	Tumor immune microenvironment	Oxidative phosphorylation	Extracellular vesicles	Lipopolysaccharide
10	Immune checkpoint inhibitors	Tumor microenvironment	Microrna	Reactive oxygen species

**FIGURE 7:**
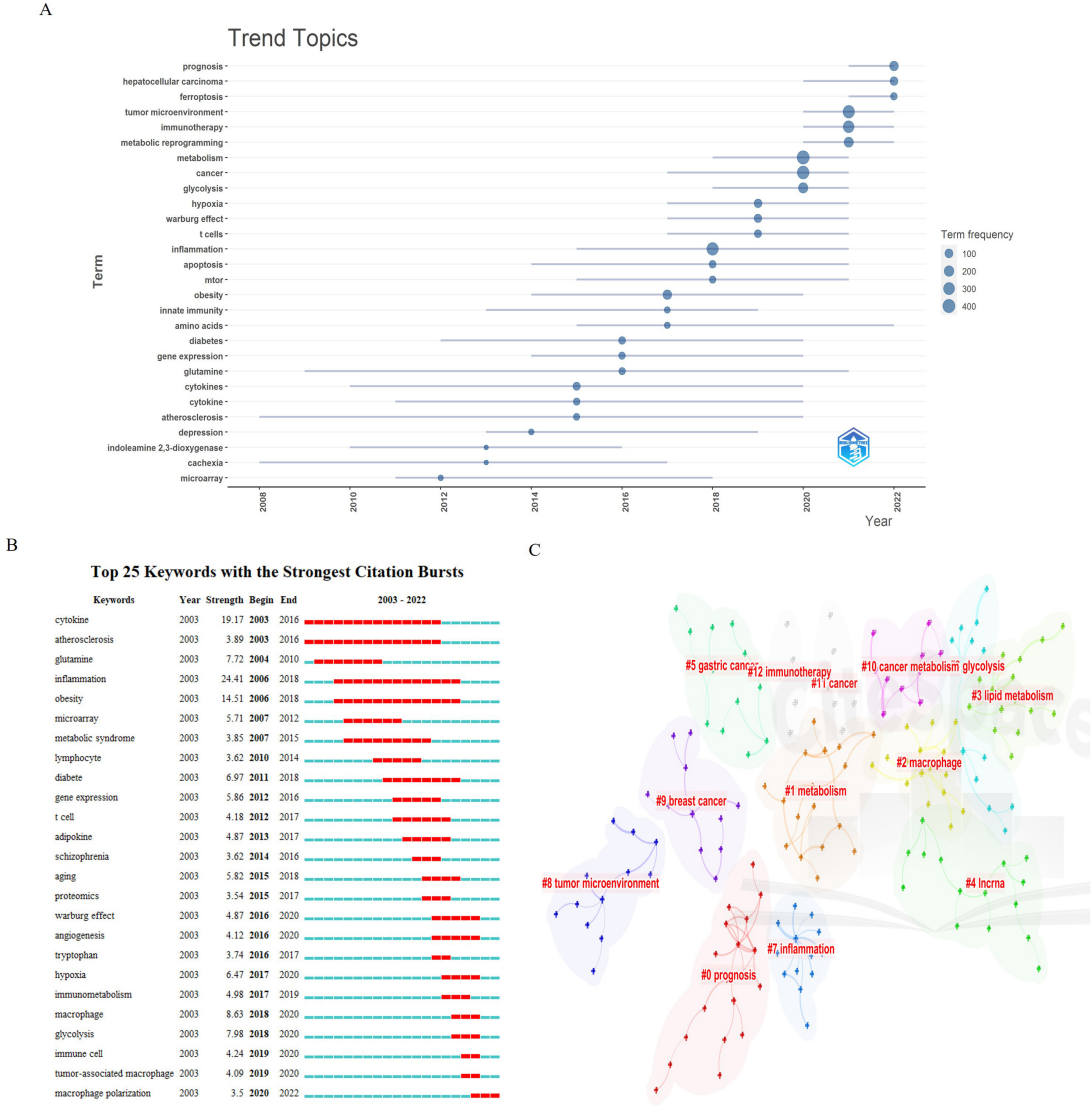
Keyword occurrence and burst analysis. (A) Trend topics based on author's keywords over time using R‐bibliometric; (B) top 25 author's keywords with the strongest citation bursts using Citespace; (C) co‐occurrence clusters of author's keywords using Citespace.

Notably, recent years have witnessed the emergence of high‐frequency keywords such as “tumor microenvironment,” “metabolic reprogramming,” “immunotherapy,” and “ferroptosis” (Figure 7A). Furthermore, the keyword burst map generated by CiteSpace also revealed a shifting focus of the study towards the following themes: “immunometabolism,” “glycolysis,” “immune cell,” “tumor‐associated macrophage,” and “macrophage polarization” (Figure 7B). Additionally, a timeline visualization of keyword clustering was presented using CiteSpace (Figure 7C), highlighting the hotspots of this study. Macrophages, glycolysis, TME, cancer metabolism, and immunotherapy continued to be hot topics in 2022.

## 4 Discussion

### 4.1 Summary of Findings

In this study, we performed a visualization‐based bibliometric analysis of 4726 articles related to metabolic reprogramming and the TME from the WoSCC database. The results indicate that the annual number of publications is on the rise. Since 2016, there has been substantial growth in the number of articles in this field, as evidenced by trends in annual publications and total citations. Since Hanahan and Weinberg [26] summarized the two phenomena of immune escape and metabolic reprogramming as emerging hallmarks of cancer in 2011, the number of articles in this field has grown explosively. Although cancer cells opt for metabolic alterations to support their rapid growth and survival in the TME, there is a growing appreciation that tumor‐associated immune cells within the TME also undergo metabolic reprogramming, which influences their tumor‐promoting or tumor‐suppressing properties [30]. Owing to the critical role of immune cells in tumor progression, several bibliometric studies on different immune cell types, including T‐cells and tumor‐associated macrophages (TAMs), have been reported [31, 32]. Studies on this topic are ongoing and have shown an overall upward trend.

Analysis of the most prolific journals showed that *Frontiers in Immunology*, *Frontiers in Oncology*, and *Cancers* obtained the top three highest degrees of productivity, indicating the long‐standing interest and role of these journals in metabolic reprogramming‐TME‐related research. In contrast, the top three most cited academic journals were *Nature*, *Cancer Research*, and *Cell* in the field. Notably, highly productive periodicals are not high‐quality or influential journals on these topics, which are mainly driven by the number of publications in these journals.

We also analyzed the most influential countries and institutions in the fields of metabolic reprogramming and the TME. The United States was the leading country in terms of contributions to this field, as evidenced by the United States obtaining its highest number of publications, citations, and international collaborative relationships. This indicates that the United States has a significant advantage over other countries in terms of academic influence. Moreover, among the top 20 productive institutions, eight are in the United States, which is the main research center in this field and has the strongest international collaborative network. Notably, 90% of the 20 most productive institutions are located in China. This demonstrates that China, as a developing country, is making progress in scientific research and is gradually narrowing the gap with the country's investment in funding.

According to the authors' analysis, Ho Ping‐Chih was the most productive author and had extensive collaboration with other scholars focusing on the role of metabolic reprogramming in immunosuppressive TME. In 2017, the author published two articles on the immunomodulatory function of T cells in the TME [33, 34]. Thereafter, this scholar published a series of articles on immunometabolic modulation and anti‐tumor immunity in prestigious professional journals. Highly productive author in this field will provide new therapeutic opportunities for improving antitumor responses. Furthermore, Pearce, Edward J. was the most cited author, who focused on immunometabolism for 16 years since publishing their first in 2007. This suggests that his teams are potential academic collaborators for researchers.

Keyword analysis revealed that the most frequently appearing keywords were related to TME and metabolic reprogramming, indicating that they were current research hotspots. In the keyword co‐occurrence network diagram, all keywords were categorized into several popular research directions, focusing on tumor immune environment, metabolic reprogramming, immunometabolism, and immunotherapy. The nodes of the author's keywords drew attention to emerging topics, including tumor‐associated immune cells, prognosis, glycolysis, and ferroptosis. Based on these keywords, we will further elaborate on these topics in the fields of TME and metabolic reprogramming.

### 4.2 Tumor Microenvironment and Immunotherapy

Immunotherapies, including immune checkpoint inhibitors (ICBs) and chimeric antigen receptor cells, have revolutionized cancer treatment and revitalized tumor immunology [35]. Clinical trials of ICBs have shown unprecedented, sustained responses, albeit in a subset of patients. In contrast to conventional therapies, immunotherapy triggers anti‐tumor responses by acting on the immune system. A positive response to immunotherapy usually depends on the interaction between tumor cells and immune regulation within the TME, which plays an important role in suppressing or enhancing immune responses [36]. Programmed cell death receptor (PD‐1) and cytotoxic T‐lymphocyte‐associated protein 4 (CTLA‐4) can inhibit effector T cells [37]. Programmed cell death‐ligand 1 (PD‐L1) induces apoptosis of anti‐tumor T lymphocytes and promotes tumor growth. Thus, PD‐1 binds to its ligand and induce inhibitory signals that reduce T‐cell proliferation. PD‐1 receptor inhibition can overcome immune resistance in TME. Binding of CTLA‐4 to B7 inhibits T cell activation. Therefore, blocking the immune checkpoint B7/CTLA‐4 enhances the activation of tumor‐specific T cells.

Although immunotherapy research focusses primarily on T cells, there is growing evidence that B lymphocytes have crucial synergistic roles in tumor suppression [38, 39]. Tumor‐infiltrating B lymphocytes (TIL‐Bs) are a major component of tertiary lymphoid structures in tumor tissues and exhibit anti‐tumor functions, mainly by producing cytokines and antibodies [40]. B lymphocytes differentiate into plasma cells and produce tumor‐specific antibodies, which mark tumor cells for destruction or recruit other immune cells to eliminate them [41]. The interactions between B lymphocytes and other immune cells in the TME are essential for an effective antitumor immune response, highlighting the interlinkage of immunemetabolism in anti‐tumor therapy. TIL‐Bs have strong prognostic significance and are emerging as key predictors of ICBs response in human cancers [42]. Recently, increasing numbers of TIL‐Bs correlate with higher survival and lower recurrence rates in a variety of solid tumors [43, 44]. Therefore, how TIL‐Bs and their tertiary lymphoid structures participate in immunotherapy‐driven anti‐tumor immunity is a subject of research in this field. These data support a role for B‐cells in tertiary lymphoid structures in response to ICBs in patients with metastatic melanoma and renal cancer [40]. Moreover, immunotherapies that enhance B‐cell responses could prove more effective than approaches concentrating on T‐cells, especially for malignancies that develop resistance to ICBs [45]. In an independent cohort study of 361 patients with hepatocellular carcinoma, a high density of TIL‐Bs resulted in better clinical outcomes [46]. It has also been suggested that TIL‐B accumulation plays a role in anti‐tumor immunity and has prognostic value in patients with metastatic colorectal cancer [47]. Studies in patients with breast cancer have shown that TIL‐Bs can predict better survival and treatment response [48]. CD20+ and CD8+ TIL synergistically mediate anti‐tumor immunity in ovarian cancer, thereby significantly prolonging survival, suggesting that TIL‐Bs support the T lymphocyte response to cancer [49]. B‐cell‐mediated humoral immune responses play an important role in effective immunotherapy for endometrial cancer [50, 51]. Therefore, current research has focused on manipulating B‐cell responses to induce immune protection through immunotherapy, as well as modulating humoral responses to achieve synergistic immune control in human cancer [49, 52].

TAMs are among the most popular immune cell subsets in TME research. An increasing number of studies have shown that TAMs have a series of functions that promote tumor development, such as supporting tumor cell proliferation, invasion, and metastasis, and are highly correlated with poor prognosis in cancer patients [53]. For example, M2‐type TAMs mediate tumor immune escape by inhibiting T cell tumor‐killing effects through various pathways, and promoting tumor cell neovascularization by secreting vascular endothelial growth factors and other factors [54]. Therefore, reshaping the immunophenotype and function of TAMs and reversing the tumor immunosuppressive microenvironment are important for the prevention and treatment of tumors.

### 4.3 Metabolic Reprogramming

**FIGURE 8:**
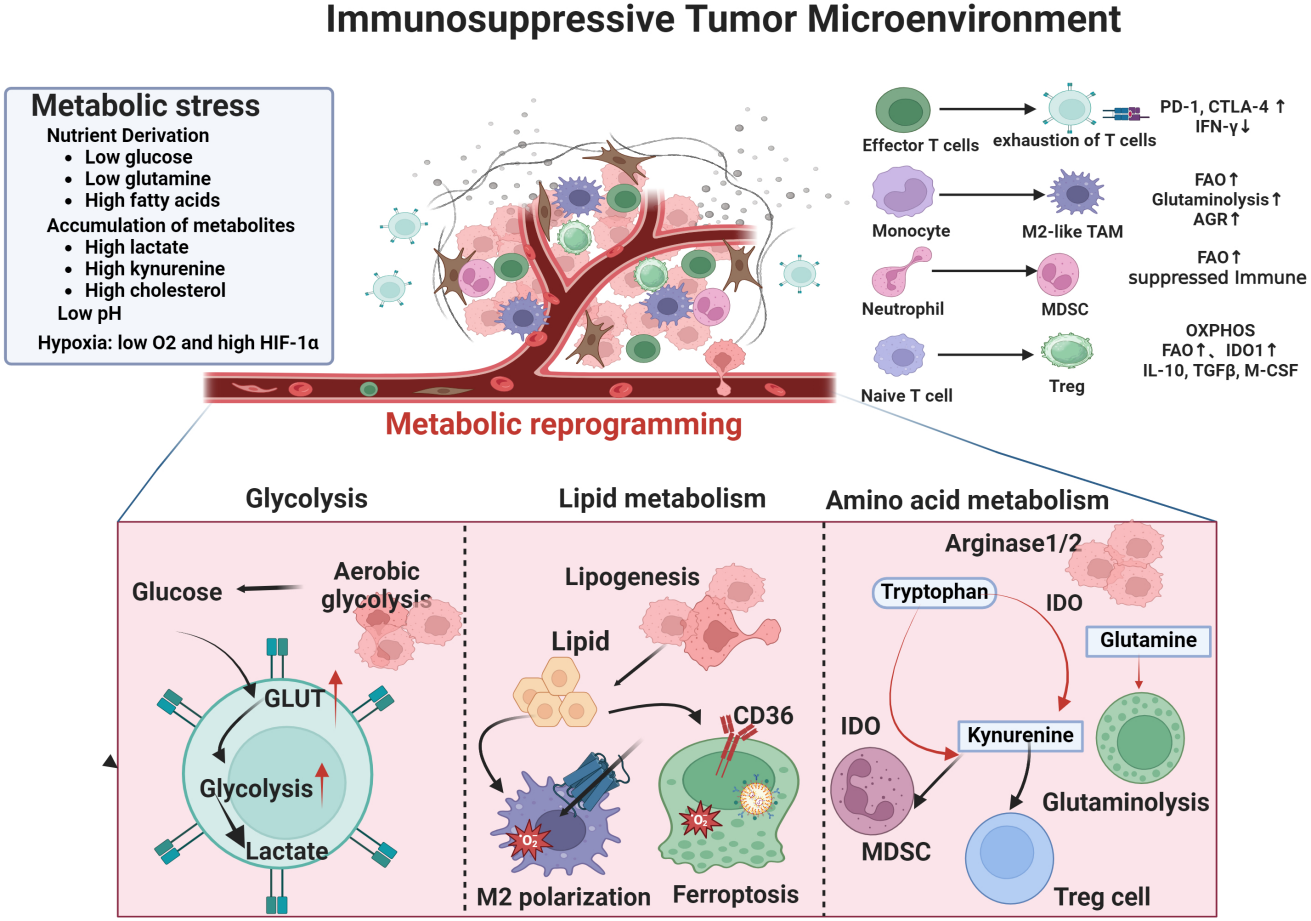
Schematic diagram of the role of metabolic reprogramming in the tumor immunosuppressive microenvironment. The TME is the environment that is essential for the survival of tumors and is often characteristic of low‐oxygen, low‐pH, nutrient competition, and accumulation of metabolites. Such conditions often affect the effector function and differentiation of immune cells, including T effector cell exhaustion, M2‐like macrophages, and more Treg. Consequently, these changes lead to immunosuppressive or tolerogenic phenotypes of immune cells in the microenvironment and induce metabolic reprogramming to meet energy demands. The enhanced aerobic glycolysis of tumor cells severely impedes the proliferation and effector function of CD8+ T cells. Moreover, tumor cells undergo increased lipogenesis and generate large quantities of lipids in the TME. These lipids can be absorbed by immune cells through lipid transporters, such as CD36, leading to increased lipid metabolism, heightened oxidative stress, impaired T‐cell function, and induction of ferroptosis. Furthermore, tumor cells compete with immune cells for amino acids such as asparagine and glutamine in the TME. The competition‐caused deficiency of some amino acids leads to T effector cell exhaustion. Although IDO facilitates the conversion of tryptophan into kynurenine, promoting the generation of Treg cells and myeloid‐derived suppressor cells. The above metabolic alterations inhibit tumor‐infiltrating immune cell functions at different degrees, further enhancing the suppressive immune microenvironment that affects anti‐tumor immune responses.

Tumor‐infiltrating immune cells can detect dramatic changes in the TME and undergo metabolic reprogramming [10]. Metabolic reprogramming of tumor and immune cells involves alterations in glucose, lipid, and amino acid metabolism, which may promote the immunosuppressive TME and tumor immune escape. Metabolic reprogramming is one of the most prominent features of tumor progression within the TME. We explored the impact of tumor metabolic reprogramming on the anti‐tumor immune response in the TME from the following viewpoints (Figure 8).

Aerobic glycolysis is an inefficient way to generate energy and consumes a large amount of glucose, which significantly affects the anti‐tumor response of immune cells [55]. Moreover, glycolysis can produces large amounts of metabolites, leading to lactate accumulation and TME acidification. This further imposes metabolic stress on tumor‐infiltrating immune cells, hinders extracellular transport of lactate of cytotoxic T cells and natural killer cells, and leads to impaired cytotoxic function [56]. Enhanced aerobic glycolysis in cancer cells and the subsequent TME acidification inhibit immune cell functionality, thereby impairing cancer immunosurveillance and facilitating tumor immune escape. Simultaneously, the glucose metabolism pattern of immune cells significantly affected cancer immunity in the TME [57, 58]. Exhausted CD8+ T cells in the TME undergo a metabolic shift towards glycolysis instead of oxidative phosphorylation, which further compromises their ability to inhibit cancer progression [59]. Therefore, cancer and immune cells undergo remarkable glycolytic reprogramming within the TME, contributing to tumor immune escape and immunosuppression.

Recently, the effect of lipid metabolism on immune cell function in the TME has become an active area of research. The accumulation of long‐chain fatty acids in the TME can trigger major transcriptional reprogramming of lipid metabolic pathways, leading to reduced fatty acid catabolism and the further induction of CD8+ T cell exhaustion [60, 61]. Cholesterol in the TME can induce an increase in fatty acid transporter CD36 expression in CD8+ T cells and then take up excessive fatty acids, which triggers lipid peroxidation and ferroptosis, resulting in the loss of T‐cell effector function and exhaustion [62, 63]. Furthermore, the inhibition of ferroptosis or CD36 expression in exhausted T cells can restore the killing ability of cytotoxic CD8+ T cells [63]. In addition, increased fatty acid content in the TME favors the generation of Tregs, which exert immunosuppressive functions and induce myeloid cells to convert to immunosuppressive and anti‐inflammatory phenotypes [64]. Collectively, abnormal lipid metabolism in the TME affects the recruitment and effector functions of tumor‐related immune cells, thereby blocking anti‐tumor immunity.

Tumor‐infiltrating cells use amino acids to promote their proliferation and invasion, and the abnormal uptake of amino acids can promote tumor immune evasion. The deficiency of certain amino acids and their metabolites in the TME can inhibit the effector functions of immune cells, especially the activation and function of effector T cells [65, 66]. Glutamine blockade inhibits the proliferation of cancer cells by inhibiting glycolytic metabolism, and also overcomes tumor immune evasion by dismantling the tumor immunosuppressive microenvironment [67]. In addition to glutamine, arginine, and tryptophan metabolism in the TME are crucial in the immune regulation of tumor cells. Indoleamine‐2,3‐dioxygenase 1, which is highly expressed in TAMs and dendritic cells of the TME, catabolizes tryptophan into kynurenine and promotes the differentiation of Tregs, thereby inhibiting the anti‐tumor immune response [68, 69]. Alterations in amino acid metabolism in the TME are closely related to the dysfunction of effector immune cells.

In view of the above, anticancer immune responses could be enhanced by targeting metabolic reprogramming in the TME. Emerging studies have developed novel adjuvant immune therapeutic modalities to enhance cancer immunotherapy by altering glucose, lipid, and amino acid metabolism [29]. For example, immunotherapeutic drugs based on glucose metabolism have been designed and developed to target key metabolic enzymes such as hexokinase and lactatedehydrogenase A [70, 71]. Acetyl‐CoA acetyltransferase 1 (ACAT1) is an enzyme that synthesizes cholesterol esters in CD8+ T cells. The ACAT inhibitor avasimibe improves the anti‐tumor immune function of CD8+ T cells and achieves better therapeutic efficacy when combined with anti‐PD‐1 immunotherapy [72]. Glutaminase controls the first step of glutamine metabolism. GLS inhibitors such as CB‐839 haves advanced to clinical trials as components of combination therapies [73]. Currently, IDO inhibitors, such as epacadostat, indoximod, and navoximod, are often used in combination with chemotherapeutic drugs or other ICBs [74].

### 4.4 Limitations

This study is the first to characterize the most frequently cited articles related to metabolic reprogramming and the TME over the past two decades through a relatively comprehensive bibliometric visualization. Bibliometrics objectively examines a research field to identify mainstream directions and emerging hotspots for more meaningful studies. This study shows that metabolic reprogramming is highly promising for regulating the immune microenvironment and response to cancer immunotherapy. The intricate interaction between the immune response and metabolic reprogramming in the TME deserves significant attention to advance treatment options for patients with cancer. However, this study has some limitations. First, the data were derived only from the WoSCC database and English publications, which may have led to bias in the results. Nevertheless, the WoSCC database is extensively recognized and widely used for bibliometric analysis, offering a substantial volume of articles that accurately depict the current state of research in this field. In addition, some high‐impact articles published in recent years have not received the attention they deserve because of their limited time span. Nonetheless, we searched and cited as much high‐quality recent literature as possible in the discussion. Finally, the data were obtained using bibliometric analysis methods, which may have led to bias discussed in other bibliometric studies [21, 32]. However, our study is broadly consistent with the most recent conventional reviews while providing researchers with more objective data and implications.

## 5 Conclusion

In conclusion, this study provides a thorough analysis of the research trends and hotspots of the TME and metabolic reprogramming over the past two decades. The TME characteristics are important factors affecting the efficacy of anti‐tumor immunotherapy. Metabolic reprogramming of tumor and immune cells plays a pivotal role in regulating the immune microenvironment and response to cancer immunotherapy. Future research is necessary to untangle the intricate interaction between metabolic reprogramming and the TME, and gaining a deeper understanding will be advantageous in advancing treatment options for cancer patients.

## Author Contributions

**Yupeng Xi:** data curation (lead), funding acquisition (supporting), investigation (lead), writing – original draft (lead). **Rui Liu:** data curation (equal), investigation (equal), writing – review and editing (equal). **Xing Zhang:** data curation (equal), methodology (lead), software (lead). **Qiujun Guo:** data curation (equal), methodology (equal), visualization (lead). **Xiwen Zhang:** methodology (equal), validation (lead). **Zizhen Yang:** methodology (equal), validation (equal). **Honggang Zheng:** project administration (lead), supervision (equal), writing – review and editing (equal). **Qingqiao Song:** conceptualization (equal), supervision (lead), writing – review and editing (equal). **Baojin Hua:** conceptualization (lead), supervision (equal), writing – review and editing (lead).

## Conflicts of Interest

The authors declare no conflicts of interest.

## Data Availability

The original data supporting the conclusions of this manuscript will be made available by the corresponding authors, without undue reservation.
